# Germanium: a new catalyst for diamond synthesis and a new optically active impurity in diamond

**DOI:** 10.1038/srep14789

**Published:** 2015-10-05

**Authors:** Yuri N. Palyanov, Igor N. Kupriyanov, Yuri M. Borzdov, Nikolay V. Surovtsev

**Affiliations:** 1 Sobolev Institute of Geology and Mineralogy, Siberian Branch of Russian Academy of Sciences, Koptyug ave 3, Novosibirsk, 630090, Russia; 2 Novosibirsk State University, Novosibirsk, 630090, Russia; 3 Institute of Automation and Electrometry, SB RAS, Koptyug ave 1, Novosibirsk, 630090, Russia

## Abstract

Diamond attracts considerable attention as a versatile and technologically useful material. For many demanding applications, such as recently emerged quantum optics and sensing, it is important to develop new routes for fabrication of diamond containing defects with specific optical, electronic and magnetic properties. Here we report on successful synthesis of diamond from a germanium-carbon system at conditions of 7 GPa and 1,500–1,800 °C. Both spontaneously nucleated diamond crystals and diamond growth layers on seeds were produced in experiments with reaction time up to 60 h. We found that diamonds synthesized in the Ge-C system contain a new optical centre with a ZPL system at 2.059 eV, which is assigned to germanium impurities. Photoluminescence from this centre is dominated by zero-phonon optical transitions even at room temperature. Our results have widened the family of non-metallic elemental catalysts for diamond synthesis and demonstrated the creation of germanium-related optical centres in diamond.

Advances in diamond synthesis and growth techniques have paved the way for this unique material to many existent and prospective applications^1^,^2^,^3^,^4^. At present a great deal of research and development on diamond has been focused on the engineering of colour centres with specific properties^5^,^6^. To greater extent, this subject has been stimulated by the prospects of diamond application in various opto-electronic devices^1^,^2^ and recently emerged quantum technologies^7^. A vibrant example of such research and development concerns the nitrogen-vacancy centres in diamond, which are proving to be a promising system for quantum information processing^8^,^9^, nanoscale magnetomentry^10^, and biolabelling^11^. At the same time, search for new optical and paramagnetic centres in diamond relevant to these applications represents a challenging task. Recently silicon-vacancy centres have been demonstrated to possess characteristics promising for single-photon applications^12^. In addition, attempts at creating optical centres related to other impurities in diamond, such as Ni^13^, Cr^14^ and Eu^15^ have been reported.

Chemical vapour deposition (CVD) and high pressure high temperature (HPHT) techniques are commonly utilized to produce diamonds with specific properties. Modern HPHT technologies, based on group VIII transition metal solvent-catalysts, enable production of synthetic diamond grits and growing large diamond single crystals^4^,^16^. Non-metallic solvent-catalysts, representing another class of agents for diamond formation, have been introduced in early 1990s^17^. As a result of extensive investigations performed since that time a large number of substances were found to catalyze diamond synthesis at HPHT conditions, including carbonates^17^,^18^, sulphates^19^, nitrides^20^, and elemental agents such as phosphorus^21^, sulphur^22^, selenium^23^, antimony^24^, etc. Diamonds produced with non-metallic catalysts frequently exhibit unique properties related to the specificity of the catalyst composition, for instance, phosphorus doped diamonds produced in the P-C system^25^ or superconducting diamonds synthesized in the B–C system^26^. This stimulates further search for new substances capable of converting graphite to diamond. From the analysis of the existing literature it follows that there are no detailed studies on diamond synthesis using group IV elements such as silicon and germanium as the catalysts. The use of silicon as a catalyst for the conversion of graphite to diamond is obstructed by its ability to form carbide phases. Carbide forming materials may become catalysts at temperatures where they decompose^27^,^28^. But, at pressures of 5–8 GPa, which are typically employed for diamond synthesis, silicon carbide melts at temperatures as high as 2500–2600 °C^29^, making experiments on diamond synthesis in the Si-C systems highly complicated. Germanium unlike silicon does not form known carbide phases and having relatively low melting temperature (650 °C at 7 GPa) deserves a detailed investigation. To the best of our knowledge there is only one study^30^ reported very slight diamond growth in the Ge-C system at 6 GPa and 1,600 °C for 10 h, with the new layers grown on the seed crystals being less than 1 μm thick. The ability of Ge to catalyse diamond synthesis via spontaneous nucleation has not been established. Furthermore, no information on the properties of diamonds grown from germanium has been presented so far.

In this work we investigate diamond crystallization in the Ge-C system in the temperature range of 1,500–1,800 °С at pressures of 7.0 and 6.0 GPa using a kinetic approach with reaction times reaching up to 60 h. Diamond formation via spontaneous nucleation and growth on the seed crystals is established proving the role of germanium as the catalyst for diamond formation. Spectroscopic studies of the synthesized diamonds reveal a new previously unreported optical centre with a zero-phonon line (ZPL) system at 2.059 eV, emitting spectrally narrow luminescence at room temperature. We assign this new optical centre to defects involving germanium impurities in diamond.

## Results

### Diamond synthesis

**Figure 1:**
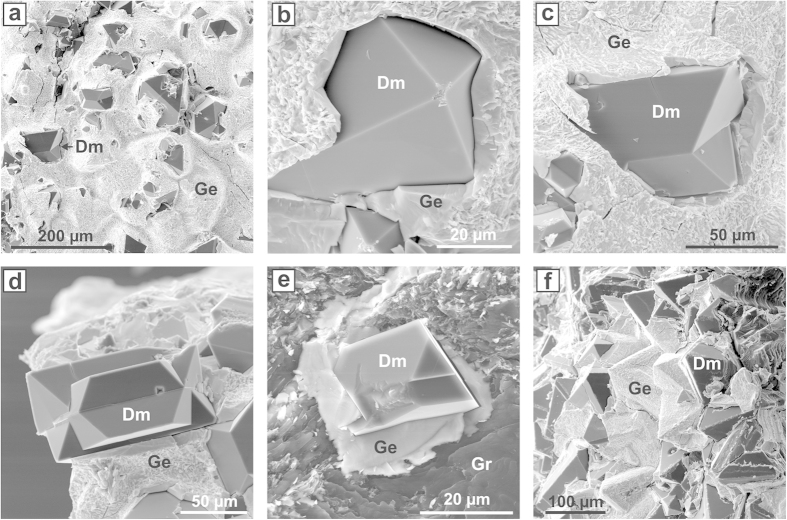
Scanning electron micrographs of diamond crystals synthesized in the germanium-carbon system. (**a**) A fragment of a quenched Ge sample with diamonds crystallized at the interface with the graphite capsule. (**b**) An octahedral diamond crystal. (**c**,**d**) Diamond crystals in the form of contact (**c**) and penetration (**d**) twins. (**e**) An octahedral diamond crystal surrounded by quenched Ge melt. (**f**) A fragment of polycrystalline diamond aggregate. Dm, Gr and Ge refer to diamond, graphite and germanium, respectively.

The conditions and results of experiments performed in the Ge-C system are summarized in Table S1 (Supplementary Information). At 7 GPa and 1,800 °C molten germanium readily reacted with graphite of the capsule giving rise to relatively intense spontaneous nucleation of diamond. In both 2-h and 5-h experiments polycrystalline diamond aggregates were produced, with the overall degree of the graphite-to-diamond transformation being approximately 30 and 90%, respectively. Diamond crystals forming the aggregates were 20–100 μm in size and exhibited only octahedral growth forms. At 1,700 °C for 1 h only slight diamond growth on the seed crystals and no spontaneously nucleated diamonds were established. Extending holding time to 5 h led to diamond crystallization via spontaneous nucleation. Approximately 10% of the initial graphite was converted to diamond. With further increases in run duration to 10 and 20 h the degree of the graphite-to-diamond transformation increased to 40 and as high as 95%, respectively. In both experiments synthesized diamonds formed polycrystalline aggregates, indicating relatively high densities of spontaneously nucleated diamond crystallization centres. At 1,600 °C the interaction between germanium melt and graphite capsule was notably weaker. No spontaneous nucleation of diamond was observed for 2 h, but only rather thin diamond growth layers were detected on the seed crystals. Spontaneously nucleated diamond in amount of approximately 80–100 crystals per mm^2^ was produced in the experiment held for 10 h. Individual octahedral crystals and twins (Fig. 1a–e) were located at the interface between the germanium melt and the graphite capsule and had sizes from 20 to 120 μm. As the reaction time was increased to 20 h the degree of the graphite-to-diamond transformation increased to 30% and synthesized diamond formed a polycrystalline aggregate (Fig. 1f). Approximately 80% of the initial graphite was converted to diamond in the experiment held for 40 h. At 1,500 °C even 20 h was not sufficient for spontaneous nucleation of diamond, though new diamond growth layers were detected on the seeds crystals. Nevertheless, extending run duration to 40–60 h we established diamond synthesis. Diamond nucleation took place at the graphite-melt interface in amount of approximately 50 crystals per mm^2^. The produced octahedral crystals and twins were up to 100–150 μm in size. To examine the effect of pressure on diamond crystallization in the Ge-C system a 40-h experiment was performed at 6.0 GPa and 1,600 °C. Both diamond spontaneous nucleation and growth on seeds was established. However, the degree of the graphite-to-diamond transformation was as low as 2%.

**Figure 2:**
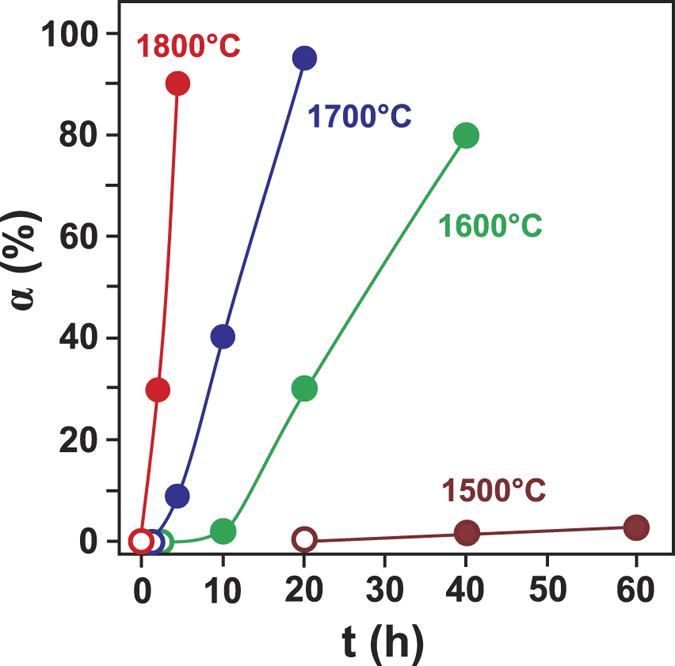
Kinetics of diamond crystallization. Degree of the graphite-to-diamond transformation (α, %) is plotted versus reaction time (t, h) for experiments at 7 GPa and 1,500–1,800 °C. Open symbols refer to experiments where no spontaneous diamond nucleation was observed.

The established features of diamond crystallization in the Ge-C system can be summarized as follows. Diamond crystals nucleate at the interface between the germanium melt and the graphite capsule. The crystals are surrounded by a germanium layer about 5–10 μm thick, through which carbon atoms diffuse and precipitate as diamond. Over the entire range of the synthesis temperatures the produced crystals shows only octahedral growth forms and are represented by octahedrons, contact and penetration twins, and irregular overgrowths (Fig. 1). The rate of diamond nucleation and growth in the Ge-C system strongly depends on the synthesis temperature. At 7 GPa the minimum temperature of diamond synthesis is 1,500 °C. Another important factor controlling diamond crystallization is the reaction time. The effect of kinetics becomes predominant at lower synthesis temperatures. The established dependence of the degree of the graphite-to-diamond transformation on the temperature and reaction time is presented in Fig. 2.

### Spectroscopic characterization of the synthesized diamonds

Fourier transform infrared absorption measurements were performed for a number of diamond crystals synthesized in the Ge-C system at different temperatures. Despite relatively small sizes of the samples, in most cases it was possible to establish that the absorption in the defect-induced one-phonon-region (1,400–800 cm^−1^) was weak compared to the intrinsic multiphonon absorption of diamond. This indicates low concentrations of impurities, first of all nitrogen, in the synthesized diamonds.

**Figure 3:**
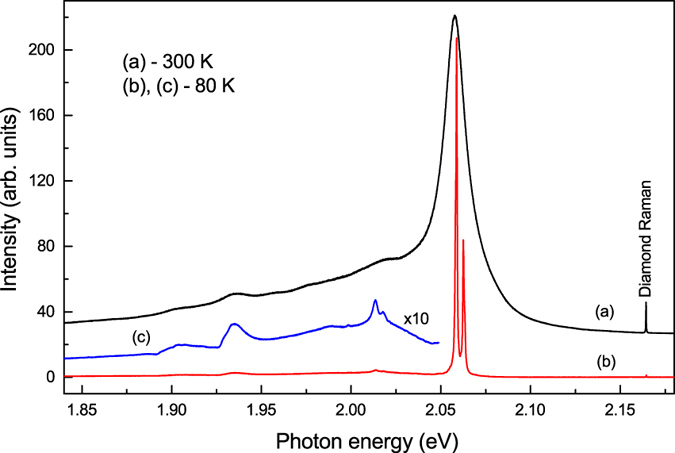
Typical photoluminescence spectra of diamonds synthesized in the Ge-C system. Spectra were recorded at (**a**) 300 K and (**b**,**c**) 80 K. Spectrum (**c**), multiplied by a factor of 10, shows details of the phonon sideband of the 2.059 eV optical system. The spectra were displaced vertically for clarity.

**Figure 4:**
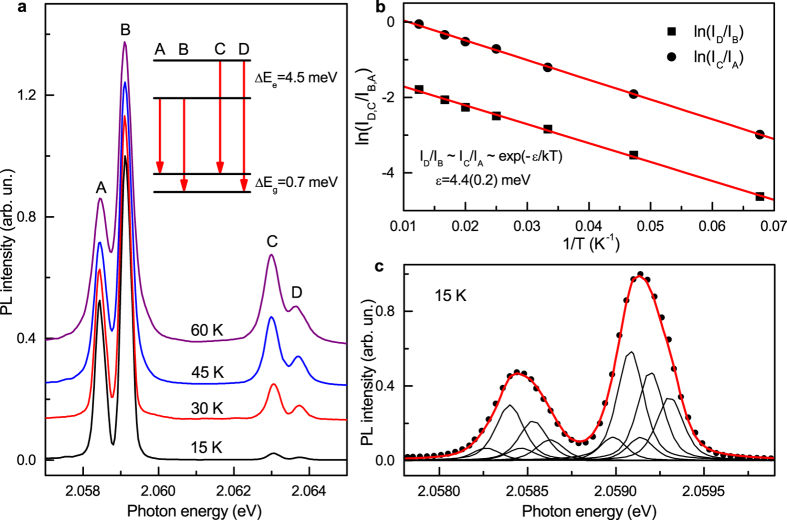
Spectroscopic characteristics of the 2.059 eV optical centre. (**a**) Photoluminescence spectra measured at different temperatures. Letters A–D are used to label the components of the ZPL system. The spectra are normalized to the intensity of line B and shifted vertically for clarity. Inset shows a proposed energy-level diagram. ΔE_g_ and ΔE_e_ are the spectral energy separations for the ground and excited states, respectively. (**b**) Logarithmic plot of ratios of luminescence line intensities ln(I_D_/I_B_) (squares) and ln(I_C_/I_A_) (circles) versus 1/T. Lines through the data points are least-squares linear fits, which give activation energy ε of 4.4 ± 0.2 meV. This value is in good agreement with the observed spectroscopic splitting in the excited state. (**c**) Photoluminescence spectrum recorded at 15 K (dots) showing asymmetric shape of zero-phonon lines A (2.0854 eV) and B (2.0591 eV). Curve through the data points is a fit to five peaks for each line. The fitting peaks are pseudo-Voigt functions and their relative intensities are fixed to the ratio of the five stable Ge isotopes abundances.

Photoluminescence (PL) measurements show that a new previously unreported system with a ZPL system at approximately 2.059 eV dominate in the spectra (Fig. 3). Occasionally it is accompanied by weak PL bands due to the well-known nitrogen-vacancy (N-V) centres 1.945 and 2.156 eV. As a rule, crystals produced at 1,600 and 1,700 °C showed higher intensities of photoluminescence (relative to the first-order Raman peak of diamond) from the 2.059 eV system. In the room temperature spectra the 2.059 eV system appears as a relatively narrow zero-phonon peak (linewidth of 4–5 nm) with a tail at lower photon energies presumably due to phonon-assisted transitions. Spectra recorded at 80 K reveal that the 2.059 eV ZPL system consists of two peaks separated by 4.5 meV. The phonon sideband accompanying the 2.059 eV ZPL system has very low intensity indicating weak electron-phonon coupling. Estimation of the Huang-Rhys factor gives a value of approximately 0.5. Two prominent peaks in the sideband lie at 2.014 and 1.935 eV (Fig. 3c) and can be assigned to phonons with wavenumbers of approximately 360 and 1000 cm^−1^. At temperatures below 80 K each component of the ZPL doublet further splits into two lines separated by about 0.7 meV (Fig. 4a). From the temperature dependence of the relative intensities of the lines comprising the 2.059 eV ZPL system (labelled in Fig. 4 as A, B, C and D) it follows that the 4.5-meV doublet structure arises from the splitting in the excited state involved in the electronic transitions (Fig. 4b). Due to small sizes of the synthesized diamond crystals we were not able to measure and analyse absorption spectra for the 2.059 eV system. Nevertheless it is very reasonable to suppose that the 0.7-meV splitting observed for each component of the 2.059 eV ZPL doublet is due to the corresponding splitting in the ground state of the centre. This idea is supported by the fact that the relative intensities of lines constituting the 0.7-meV doublets do not change with temperature. The proposed energy-level diagram for the 2.059 eV centre is shown in the inset of Fig. 4a. Although further more detailed investigations into the spectroscopic properties and nature of the 2.059 eV centre are necessary and currently underway, we can suggest that this new optical centre is related to a defect involving germanium impurity. A proof for this assignment could be observation of the isotopic splitting of the zero-phonon lines. Natural germanium has five stable isotopes in the abundance ratios [^70^Ge]:[^72^Ge]:[^73^Ge]:[^74^Ge]:[^76^Ge] = 20.8:27.5:7.7:36.3:7.6. With such a rich composition of isotopes and assuming possible effects of inhomogeneous broadening it is likely that the individual isotope-split line components would be difficult to observe. We however note that in the spectra recorded at 15 K, zero-phonon lines at 2.0584 (line A) and 2.0591 (line B) eV have a clear asymmetric shape (Fig. 4c). This asymmetry may possibly be a consequence of unresolved isotopic splitting. As demonstrated in Fig. 4c both lines can be reasonably well fitted with five peaks, whose relative intensities correspond to the abundances of germanium isotopes (see Supplementary Information for more details). Obviously, this fit provides only one possible explanation of the observed asymmetry of the zero-phonon lines and cannot be considered as proof of the isotopic splitting. Diamond synthesis experiments with isotopically enriched germanium are necessary to prove this hypothesis.

**Figure 5:**
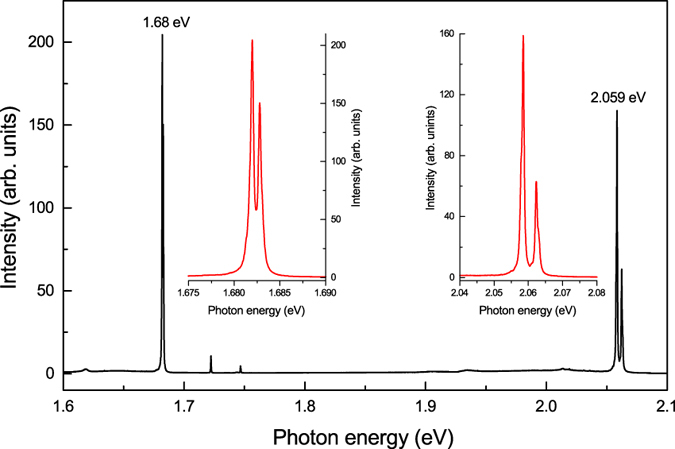
Typical photoluminescence spectrum of diamonds synthesized in the Mg_0.9_-Ge_0.1_-C system. The spectrum was recorded at 80 K with the 532-nm excitation for a diamond crystal produced in the Mg_0.9_-Ge_0.1_-C system at 7 GPa and 1700 °C. The insets show ZPL regions of the 2.059 and 1.68 eV centres.

To get further support for the assignment of the 2.059 eV centre to a defect involving germanium impurities in diamond, we performed several experiments on diamond synthesis in an Mg-Ge-C system. Recently we have undertaken a detailed study on diamond crystallization in the Mg-C system at HPHT conditions^31^. It has been found that one of the specific features of the diamond crystals produced in the Mg-C system is the abundance of the silicon-vacancy (Si-V) optical centres (1.68 eV), with silicon being present as a trace impurity in the starting materials. Supposing that growth conditions favouring incorporation of Si in diamond would also favour incorporation of Ge, we selected an Mg_0.9_-Ge_0.1_-C system for the test experiments. A detailed account of diamond crystallization in this system will be given elsewhere. Here we present photoluminescence spectra recorded for the synthesized diamond crystals (Fig. 5). The spectra are quite spectacular exhibiting both the 1.68 eV Si-V centres and the 2.059 eV centres as the dominant PL features. These results provide additional support to our conclusion that the observed 2.059 eV centres are related to germanium impurities in diamond.

It is now worth noting that the 2.059 eV optical centre resembles in many respects the well-known 1.68 eV silicon-vacancy centre, suggesting some structural similarities for these defects. Indeed, both centres are characterized by low magnitude of the electron-phonon coupling, resulting in intense emission from zero-phonon lines even at room temperature. In both cases the ZPL system consists in first approximation of four lines, originating from electronic transitions between the ground and excited states both split in doublets. As is known, the silicon-related 1.68 eV centre comprises a silicon atom located in the so-called double semi-vacancy position^32^,^33^. Germanium has a similar outer shell electronic structure as silicon, and if incorporated in diamond may produce defects with similar structure and electronic configuration. The covalent radius of germanium (122 pm) is somewhat larger than that of silicon (111 pm), making its incorporation in diamond less efficient. On the other hand, there are known examples of elements with similar (Ni, 121 pm) or even larger (Co, 126 pm) covalent radii, that are known to incorporate in the diamond lattice as point defects^34^. Theoretical calculations reported by Goss *et al.*^35^, predict that germanium-vacancy complexes in diamond possess a double semi-vacancy structure with D_3d_ symmetry and have formation and binding energies similar to those of the silicon-vacancy defects. For the 1.68 eV Si-V centre it has been recently shown that splitting of the degenerate states involved in the optical transitions is mainly due to spin-orbit interaction^33^. Since germanium has larger spin-orbit coupling constants than silicon, larger splitting of the degenerate electronic states can be expected, as we indeed observed for the 2.059 eV centre.

The concentration of atomically dispersed Ge impurities in diamonds synthesized in this study is difficult to determine accurately using techniques for trace element analysis. This primarily is due to the occurrence of small micron-sized inclusions of the germanium solvent-catalyst (see Supplementary Information Section B). As follows from preliminary analyses made by the Laser Ablation Inductively Coupled Plasma Mass Spectrometry (LA-ICP-MS) and Wavelength Dispersive X-ray Spectroscopy (WDS) (see Supplementary Information Section D), the concentration of Ge point defects in diamond is likely to be at or below parts per million level.

## Discussion

In summary, we have experimentally demonstrated the catalytic ability of germanium for the conversion of graphite to diamond at HPHT conditions. Unlike the previous work^30^ reported minute seeded growth of diamond from germanium, diamond crystallization via spontaneous nucleation and growth is achieved in the present study with the overall degree of the graphite-to-diamond conversion being as high as 90–95%. It is found that diamond crystallization takes place from carbon solution in germanium melt via the mechanisms similar to those established for many other metal and non-metallic catalysts^4^. Temperature and reaction time are the main parameters controlling diamond formation in the germanium melt. The minimal temperature of diamond synthesis considerably exceeds the melting point of germanium and is equal to 1,500 °С at 7 GPa. The effect of crystallization kinetics dominates at lower temperatures and diminishes as temperature increases. Spectroscopic studies of diamonds synthesized in the Ge-C system revealed a new previously unreported optical centre with ZPL system at 2.059 eV. Based on the data obtained we propose that the 2.059 eV centre is related to germanium impurities in diamond. The structure of the corresponding defect can tentatively be assigned to a germanium atom occupying a double semi-vacancy position. This centre has weak vibronic coupling and produces photoluminescence in relatively narrow spectral region of the ZPL even at room temperature, which makes it a promising candidate for further investigations relevant to single-photon and quantum optics applications. In view of very limited number of chemical elements able to incorporate in diamond as point defects, the identification of germanium as a new optically active impurity in diamond appears a useful step in expanding the pool of colour centres in this scientifically interesting and technologically important material.

Finally, we want to note that after this work had been carried out and submitted for consideration of publication in Scientific Reports we became aware of a recent un-refereed article^36^ reporting a novel colour centre in diamond with a ZPL at 602.7 nm (~2.06 eV). The centre was found in type IIa diamonds implanted with Ge ions and in diamond films doped with Ge during MPCVD growth. The authors assigned this centre to a germanium-vacancy (GeV) point defect in diamond, which is in good agreement with the conclusions made in the present study.

## Materials and Methods

Experiments on diamond crystallization in the Ge-C system were performed using a multi-anvil split-sphere apparatus^6^ at pressures of 6.0 and 7.0 GPa in a 1,500–1,800 °C temperature range. The experimental duration was varied from 1 to 60 h. Temperature was measured in each experiment using a PtRh30/PtRh6 thermocouple, whose junction was placed near the crystallization capsule. Details of the temperature and pressure calibration have been presented elsewhere^37^. The starting materials were a graphite rod (99.99% purity), germanium (99.999% purity) and cubooctahedral synthetic diamonds (*ca*. 0.5 mm) as the seed crystals. Graphite rods were machined into thick-walled (1.5–1.8 mm) capsules with the outer diameter of 7.0 mm and 6.5 mm high. Pressed Ge cylinders with diamond seed crystals were placed inside the graphite capsules. The capsules were protected from the outside with a 0.1 mm thick molybdenum foil. After experiments, the samples were studied using an Axio Imager.Z2m optical microscope and a LEO 420 scanning electron microscope (SEM). Formation of small diamond crystals was confirmed by Raman spectroscopy. The degree of the graphite-to-diamond conversion, α, which is defined as α = M_Dm_ / (M_Dm_ + M_Gr_) × 100, where M_Dm_ is the mass of synthesized diamond and M_Gr_ is the mass of residual graphite, was determined for each growth run. For this, samples were first treated in hot nitric acid to dissolve germanium. The obtained mixture of diamond and graphite was weighed, then graphite was dissolved in a hot mixture of K_2_Cr_2_O_7_ and H_2_SO_4_ and only produced diamond was weighed. Synthesized diamond crystals were characterized by infrared (IR) absorption and photoluminescence (PL) spectroscopy. IR spectra were recorded using a Bruker Vertex 70 Fourier transform infrared spectrometer fitted with a Hyperion 2000 microscope.

Raman/photoluminescence measurements at temperatures in the range 80–300 K were made using a Horiba J. Y. LabRAM HR800 spectrometer with a 532-nm solid state laser and a Linkam FTIR600 heating/freezing stage. Spectral resolution was of about 1.0 cm^−1^. PL spectra at temperatures in the range 15–90 K were measured using an Acton TriVista 777 triple monochromator equipped with a Princeton Instruments Spec-10 LN-cooled CCD camera and an ARS DE-204SI optical closed-cycle helium cryostat. The measurements were performed with a 532-nm solid state laser as the excitation source and spectral resolution of 0.5 cm^−1^.

Several additional experiments were performed to synthesize diamond in the Mg_0.9_Ge_0.1_-C system. The experimental procedure, sample assembly and starting reagents (magnesium and graphite) were the same to those used in the experiments with the Mg-C system^31^. Synthesis runs were performed at 7 GPa, 1600–1800 °C for 2–10 h. The produced diamond crystals were characterized by photoluminescence spectroscopy (as described above).

## Additional Information

**How to cite this article**: Palyanov, Y. N. *et al.* Germanium: a new catalyst for diamond synthesis and a new optically active impurity in diamond. *Sci. Rep.* **5**, 14789; doi: 10.1038/srep14789 (2015).

## Supplementary Material

Supplementary Information
