# Supplementary material for: Germanium: a new catalyst for diamond synthesis and a new optically active impurity in diamond
Source: Sci Rep. 2015 Oct 5;5:14789. doi: 10.1038/srep14789 (PMC4593174; doi:10.1038/srep14789)
Supplement: Supplementary Information [file srep14789-s1.pdf]

Supplementary Information

Germanium: a new catalyst for diamond synthesis and a new optically active impurity in diamond

Yuri N. Palyanov, Igor N. Kupriyanov, Yuri M. Borzdov and Nikolay V. Surovtsev

A. Diamond synthesis

Table S1 shows conditions and results of the experiments on diamond crystallization in the Ge-C system.

Table S1. Conditions and results of experiments in the Ge-C system.

Run No	P (GPa)	T (°C)	Time (h)	Diamond growth on seeds	Diamond nucleation	α (%) Gr→Dm transformation
881/7	7.0	1,800	2	+	+	30
894/1	7.0	1,800	5	+	+	90
873/1	7.0	1,700	1	+	-	0
882/7	7.0	1,700	5	+	+	10
1722/2	7.0	1,700	10	+	+	40
1726/2	7.0	1,700	20	+	+	95
1709/1	7.0	1,600	2	+	-	0
1730/2	7.0	1,600	10	+	+	5
1728/2	7.0	1,600	20	+	+	30
1763/2	7.0	1,600	40	+	+	80
1729/2	7.0	1,500	20	+	-	0
1733/1	7.0	1,500	40	+	+	<1
1311/3	7.0	1,500	60	+	+	<1
1707/2	6.0	1,600	40	+	+	2

"+" – was observed; "-" – was not observed. α is the degree of the graphite-to-diamond transformation, $\alpha = M_{Dm}/(M_{Dm} + M_{Gr})100$, where M_{Dm} is the mass of obtained diamond and M_{Gr} is the mass of residual graphite. Dm - diamond, Gr - graphite.

B. Diamond characterization

Diamond crystals produced in the Ge-C system frequently contained small micron-sized inclusions, which were identified by Raman micro-spectroscopy as germanium (Fig. S1(a)). The Raman peak measured for germanium inclusions was shifted from the normal position of the TO(Γ) Raman line of germanium at 300 cm^{-1} to about 320 cm^{-1}. This shift is apparently due to residual pressures experienced by the inclusions in the diamond host crystal. Using data on the pressure dependence of the Raman line of germanium (Olego and Cardona, 1982) the residual pressure can be estimated as approximately 5.0-5.5 GPa.

Raman spectra measured from clean inclusion-free areas of diamond crystals showed only a peak located at around 1332 cm^{-1} due to the zone-center optical phonon of diamond (Fig. S1(b)). Raman measurements performed with a spectral resolution of 0.6 cm^{-1} enabled the analysis of the diamond Raman line width to be made. Using deconvolution of the measured spectra to the Voigt profile with a fixed width of the Gaussian component (instrumental resolution, w_G=0.6 cm^{-1}), we found that the width of the Lorentzian component is within 1.5-1.6 cm^{-1}, indicating low concentrations of defects in the crystals (Surovtsev and Kupriyanov, 2015).

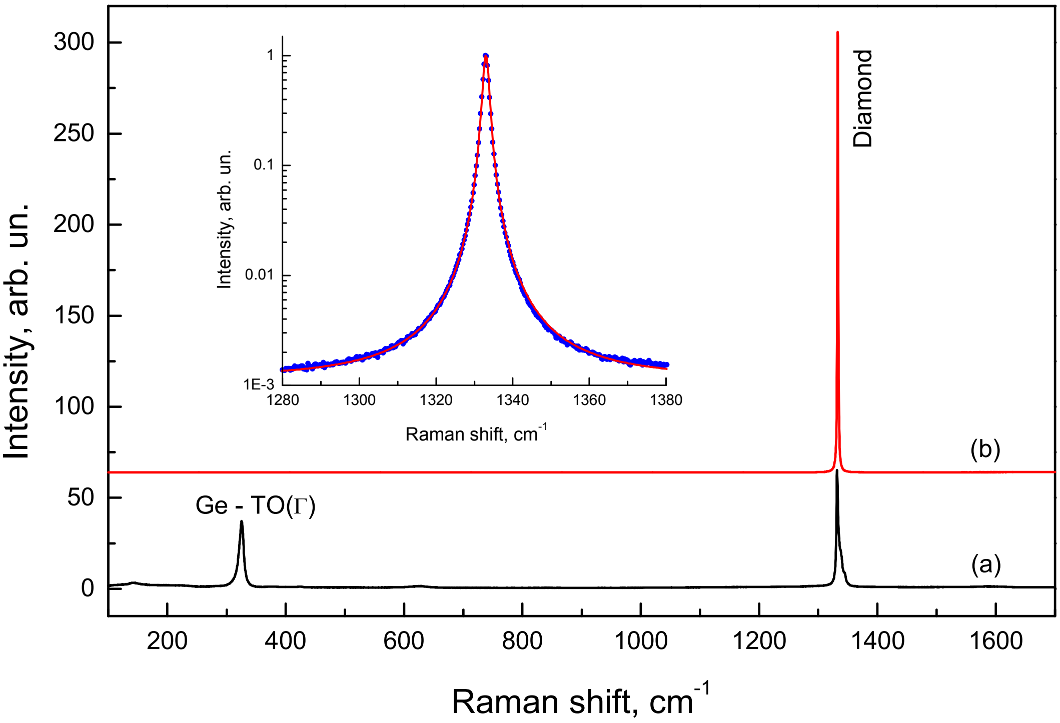

Figure S1. Raman spectra of diamonds synthesized in the Ge-C system. Spectra were recorded from a germanium inclusion (a) and from inclusion-free area of diamond (b). The spectra are shifted vertically for clarity. The inset shows the experimental Raman spectrum of diamond (blue circles) and its description with the Voigt contour with w_G=0.6 cm^{-1} and w_L=1.55 cm^{-1} (red curve).

C. Fitting of the 2.059 eV ZPLs with Ge isotopes components

Although the fit was presented solely to provide one possible explanation for the observed asymmetry of the 2.059 eV ZPLs, we suppose it reasonable to give some more details on the fitting process.

The fitting was performed with the following constraints:

(a) The relative intensities of the five peaks was fixed and set to the relative abundances of the five stable Ge isotopes, [^{70}Ge]:[^{72}Ge]:[^{73}Ge]:[^{74}Ge]:[^{76}Ge]=20.8:27.5:7.7:36.3:7.6.

(b) The distance between the peak positions on the energy scale was set so that each increase of mass by one unit produces equal shifts in the zero-phonon energy, i.e.

$$\frac{1}{2}(h\nu^{70} - h\nu^{72}) = \frac{1}{2}(h\nu^{72} - h\nu^{74}) = \frac{1}{2}(h\nu^{74} - h\nu^{76}) = h\nu^{72} - h\nu^{73} = \Delta h\nu,$$

with $\Delta h\nu$ being a free parameter.

(c) The fitting peaks were taken in the form of the pseudo-Voigt function ($V_p(x)$), which is a sum of Lorentzian ($L(x)$) and Gaussian ($G(x)$) functions and have an advantage of being analytical:

$V_p(x)=m\times L(x)+(1-m)\times G(x)$

The coefficient m was set 0.5 and both Lorentzian and Gaussian components were set to have the same width for all fitting peaks.

To sum up, there were four free parameters in the fitting process: position, intensity and width of one isotopic peak and energy separation between the peaks.

The best fit was obtained with a value of $\Delta h\nu$ of 0.06±0.01 meV. This value seems physically meaningful. For instance, for the well-known 1.40 eV Ni centre in diamond the isotopic shift of the ZPL of 0.08 meV per one unit mass has been established (Nazare et al., 1991).

D. Measurements of Ge concentration in diamond

The Ge content of the synthesized diamonds was measured using a PerkinElmer NexION 300D quadrupole mass spectrometer with inductively coupled plasma (ICP-MS) fitted with a New Wave Research 213 nm Nd:YAG UV laser ablation (LA) system. Carbon of diamond (analyzed as $^{13}C^{+}$) was used as the internal standard. The USGS microanalytical reference materials GSC-1G, GSD-1G and GSE-1G, containing 3.5, 33 and 323 wt. ppm of Ge (Jochum et al., 2005), were used as the external standards. ^{73}Ge and ^{74}Ge isotopes were analyzed. For diamonds synthesized in the Ge-C system the resulting Ge concentrations were highly irreproducible ranging from a few hundred to less than one atomic ppm. This inconsistency is apparently connected with the presence of germanium inclusions in these diamonds as described above. Diamond crystals produced in the Mg-Ge-C system were of better quality and for the LA-ICP-MS analysis it was possible to select crystals containing virtually no visible inclusions. For these diamonds we obtained more consistent Ge concentration data ranging within 0.5-5 atomic ppm. Note that the occurrence of micron-submicron sized inclusions of the solvent-catalyst ($Mg_{0.9}Ge_{0.1}$) in the analyzed diamonds still cannot be completely excluded.

The low concentration of atomically dispersed Ge impurities in diamonds synthesized in both Ge-C and Mg-Ge-C systems was confirmed by wavelength dispersive X-ray spectroscopy (WDS). Using an Oxford Instruments INCA Wave WDS spectrometer attached to a Tescan MIRA3 LMU scanning electron microscope we found that the Ge concentrations in diamond are below the detection limit (~50 ppm). Note that in this case special care was taken to ensure that the solvent-catalyst inclusions were not sampled.

References

D. Olego and M. Cardona, Pressure dependence of Raman phonons of Ge and 3C-SiC. *Phys. Rev. B* **25**, 1151-160 (1982).

N. V. Surovtsev and I. N. Kupriyanov, Temperature dependence of the Raman line width in diamond: Revisited. *J. Raman Spectrosc.* **46**, 171–176 (2015).

M. H. Nazare, A. J. Neves and G. Davies, Optical studies of the 1.40 eV Ni center in diamond. *Phys. Rev. B* **43**, 14196-14205 (1991).

K. P. Jochum, M. Willbold, I. Raczek, B. Stoll, K. Herwig, Chemical characterisation of the USGS reference glasses GSA-1G, GSC-1G, GSD-1G, GSE-1G, BCR-2G, BHVO-2G and BIR-1G using EPMA, ID-TIMS, ID-ICP-MS and LA-ICP-MS. *Geostandards and Geoanalytical Research* **29**, 285-302 (2005).
